# Initial sites of hepadnavirus integration into host genome in human hepatocytes and in the woodchuck model of hepatitis B-associated hepatocellular carcinoma

**DOI:** 10.1038/oncsis.2017.22

**Published:** 2017-04-17

**Authors:** R Chauhan, N D Churchill, P M Mulrooney-Cousins, T I Michalak

**Affiliations:** 1 Molecular Virology and Hepatology Research Group, Division of BioMedical Sciences, Health Sciences Centre, Memorial University, St John's, Newfoundland and Labrador, St John’s, NL, Canada

## Abstract

Hepatitis B virus (HBV) and the closely related woodchuck hepatitis virus (WHV) are potent carcinogens that trigger development of primary hepatocellular carcinoma (HCC). The initial sites of hepadnavirus–host genome integration, their diversity and kinetics of formation can be central to virus persistence and the initiation and progression of HCC. To recognize the nature of the very early virus–host interactions, we explored *de novo* infection of human hepatocyte-like HepaRG cells with authentic HBV and naive woodchucks with WHV. HepaRG were analyzed from several minutes post exposure to HBV onwards, whereas woodchuck liver biopsies at 1 or 3 h and 6 weeks post infection with WHV. Inverse PCR and clonal sequencing of the amplicons were applied to identify virus–host genomic junctions. HBV and WHV DNA and their replication intermediates became detectable in one hour after virus exposure. Concomitantly, HBV DNA integration into various host genes was detected. Notably, junctions of HBV X gene with retrotransposon sequences, such as LINE1 and LINE2, became prominent shortly after infection. In woodchucks, insertion of WHV X and preS sequences into host genome was evident at 1 and 3 h post infection (h.p.i.), confirming that hepadnavirus under natural conditions integrates into hepatocyte DNA soon after invasion. The HBV and WHV X gene enhancer II/core promotor sequence most often formed initial junctions with host DNA. Moreover, multiple virus–virus DNA fusions appeared from 1 h.p.i. onwards in both infected hepatocytes and woodchuck livers. In summary, HBV DNA integrates almost immediately after infection with a variety of host’s sequences, among which tandemly repeating non-coding DNAs are common. This study revealed that HBV can engage mobile genetic elements from the beginning of infection to induce pro-oncogenic perturbations throughout the host genome. Such swift virus insertion was also evident in natural hepadnaviral infection in woodchucks.

## Introduction

Hepatitis B virus (HBV) is a pro-oncogenic virus and chronic HBV infection is the main cause of primary hepatocellular carcinoma (HCC) with attributed 780 thousand deaths annually worldwide.^1, 2^ HBV carcinogenesis have been directly linked to the transforming properties of the virus X and truncated preS-S proteins and, in particular, to integration of viral DNA altering the host’s genome stability and expression of individual genes.^3, 4, 5^ The integration of HBV X (HBx) and S gene sequences had been commonly observed in HBV-associated HCC and the prevailing opinion was that the virus insertions are random throughout the liver genome. More recent high-throughput studies however indicated that certain genomic sites are more frequently targeted for HBV integration than others.^6, 7, 8^ Fusion of HBx with retrotransposon LINE1 (long-interspersed nuclear element-1 or L1) and the resulting HBx-LINE1 chimeric non-coding RNA transcripts were found in a significant portion of HBV-positive HCCs and coincided with a decreased survival of the patients.^9^ These chimeric transcripts display tumor promoting properties and may foster liver injury by sequestering hepatocellular microRNA-122.^9, 10^ It might also be that fusion of viral DNA with LINE1 may spread viral genetic material across the host’s genome owing to the intrinsic mobility of this retrotransposon.^11, 12^

The direct oncogenic role of HBV is most perceptible in HCC developing in the absence of chronic hepatitis, as in persons with asymptomatic occult HBV infection who carry HBV DNA in the absence of serum HBV surface antigen.^13, 14, 15^ This also is evident in the woodchuck model that closely mimics molecular and pathogenic events in HBV-infected humans.^16, 17^ In primary occult infection caused by very small amounts of woodchuck hepatitis virus (WHV), HCC develops in the context of normal liver histology, whereas the liver and immune system carry integrated WHV DNA.^18^ HCC also arises at a similar frequency (~20%) after acute hepatitis followed by WHV DNA-positive, virus surface antigen-negative infection, termed as secondary occult infection.^16, 19^ For comparison, the majority (~80%) of WHV DNA-positive, virus surface antigen-positive animals with chronic hepatitis develop HCC, indicating that in the context of protracted virus replication chronic inflammation augments HCC development.^16, 20, 21^

Dissection of the earliest events in HBV infection has been hampered by the lack of *in vitro* infection models. However, with the recent establishment of human hepatocyte-compatible cultures, such studies became feasible.^22, 23^ In our study, exploring HepaRG cells susceptible to authentic HBV and the woodchuck–WHV infection model, we determined the time of the appearance and the initial sites of hepadnavirus integration, and the profile of virus insertional sites across chromosomes shortly after infection. The characteristics of these very early events were unknown before but they can be critical to hepatocyte pro-oncogenic perturbations and virus lifelong persistence.

## Results

### Early hepadnaviral infection profiles in HepaRG cells and woodchucks

**Figure 1:**
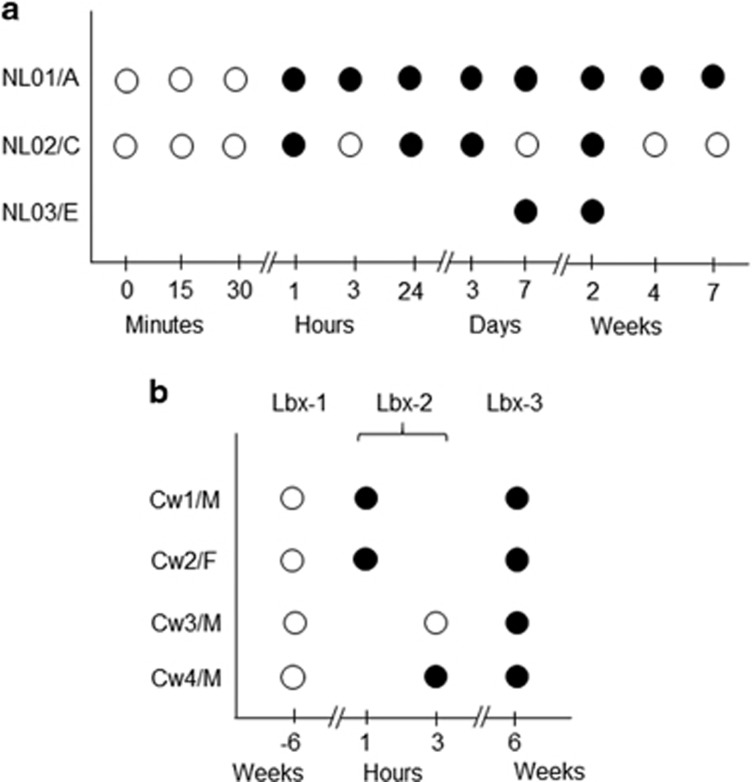
Study design and detection of HBV DNA- or WHV DNA-specific signals by invPCR/NAH after infection of HepaRG cells or healthy woodchucks. (**a**) HepaRG cells infected with authentic HBV from patients NL01.A, NL02.C and NL03.E with chronic hepatitis B. (**b**) Woodchucks Cw1 to Cw4 intravenously infected with WHVtm/3 inoculum. Liver biopsy 1 (Bx-1) obtained prior to experiment, LBx-2 at 1 or 3 h after injection with WHV, and LBx-3 acquired 6 weeks thereafter. Open circles show the acquisition time of cells or liver biopsies and closed circles represent samples in which virus DNA hybridization signals suggesting virus–host integration were detected. A, C and E, HBV genotypes; F, female; M, male.

Differentiated HepaRG cells were exposed to HBV in plasma from treatment-naive patients NL01.A, NL02.C and NL03.E with chronic hepatitis B (Table 1). The cells became HBV DNA reactive from 1 h post infection (h.p.i.) at 1.8 × 10^3^±5.7 × 10^2^ copies or virus genome equivalents (vge)/μg DNA (see Figure 1a). The virus achieved 8.8 × 10^3^±1 × 10^3^ vge/μg DNA at 1 and 2 weeks post infection (w.p.i.), and then declined until 7 w.p.i. Cells infected with NL03.E were positive at 9.5 × 10^3^±1.4 × 10^3^ vge/μg DNA at 1 and 2 w.p.i. At 14 d.p.i., HBV surface antigens were detected in ~15% of the cells by immunofluorescent staining. HBV transcripts were identified 1 h post exposure onward. HBV covalently closed circular DNA (cccDNA) became detectable from 3 d.p.i. with HBV NL01.A and NL02.C, up to 7 w.p.i. Detection of HBV RNA earlier than cccDNA was likely due to a 100-fold greater sensitivity of the HBV RNA detection assay.^18, 24^ The time-dependent kinetics of HBV infection were overall comparable to those reported before;^22, 25, 26^ however, perhaps because of the earlier time points tested and more sensitive polymerase chain reaction/nucleic acid hybridization (PCR/NAH) assays used,^18, 19, 27^ HBV DNA and its replication were detected earlier. HBV infection was not detected in HepaRG exposed to HBV for less than 1 h or after mock infection with normal human plasma (NHP).

In woodchucks, WHV DNA was detected in liver biopsies (Lbx) obtained at 1 or 3 h.p.i. (Lbx-2) and at 6 w.p.i. (Lbx-3) (see Figure 1b), as reported.^24^ WHV DNA levels were 1 × 10^5^±1 × 10^2^ vge/μg DNA in Lbx-2 and 1 × 10^8^±1 × 10^3^ vge/μg DNA in Lbx-3 samples. Liver tissue acquired prior to inoculation (Lbx-1) were WHV DNA non-reactive. WHV mRNA was identified in LBx-2 and LBx-3, whereas WHV cccDNA in LBx-3 samples.^24^

### Inv-PCR combined with NAH enhances detection of hepadnavirus–host junctions

HBV–host DNA junctions were detected by inverse-PCR (invPCR) essentially as reported.^28^ As invPCR occasionally generated products that did not contain detectable integrants after clonal sequencing, probing of the amplicons for viral DNA by NAH was implemented (Supplementary Figure 1). Consequently, only the NAH-positive amplicons were clonally sequenced. Ultimately, only virus–host fusions confirmed by sequencing were accepted. This stringent approach led to identification of HBV integrations from 1 h.p.i. to 7 w.p.i. in HepaRG exposed to HBV NL01.A (Figure 1a and Supplementary Figure 1). In the NL02.C-treated cells, the signals were also detected after 1-h outwards, whereas in those with NL03.E at 1 and 2 w.p.i. (Figure 1a). There were no NAH signals after invPCR of DNA from HepaRG collected at time zero or exposed to NL01.A or NL02.C for less an hour (Figure 1a) or to NHP.

Hybridization signals implying WHV–host junctions were detected in Lbx-2 from three woodchucks and in LBx-3 from all four animals (Figure 1b). Lbx-1 samples were non-reactive (Figure 1b).

### Hepadnavirus–host genome integration after *de novo* infection

Meticulous analysis of the virus–host junctions was performed. The coordinates of virus integrants and the right-side flanking host sequences, HBV inoculum used, and the time at which each integration was detected are identified in Table 2 and their sequence GenBank accession numbers are listed in Supplementary Table 1. Overall, there were 22 unique integration sites detected of which 14 were single hits and 8 multiple hits of the same sites. To streamline presentation, the junctions found up to 24 h.p.i. were classified as very early integration sites (VEIS), between 3 and 7 d.p.i. as early integration sites (EIS), and at 2 w.p.i. and onwards as late or not-early integration sites (NEIS). There were 9 sites identified as VEIS, 5 as EIS (with two also categorized as VEIS), and 11 as NEIS (with one also classified as EIS) (Table 2). Comparing a number of clones carrying the particular class of integrations with the total number of clones with virus–host junctions detected (*n*=76), VEIS represented one-third (31.6%), EIS one-fifth (18.4%), and NEIS a half (50%) of the clones. This suggested an increase in HBV integration events over the time examined.

WHV–host junctions discovered in woodchuck livers are presented in Table 3 and their sequence GenBank accession numbers in Supplementary Table 2. There were 10 VEIS identified in Lbx-2 collected at 1 or 3 h.p.i. and 7 classified as NEIS in Lbx-3 acquired at 6 w.p.i.

### The initial sites of HBV integration into human genome

**Figure 2:**
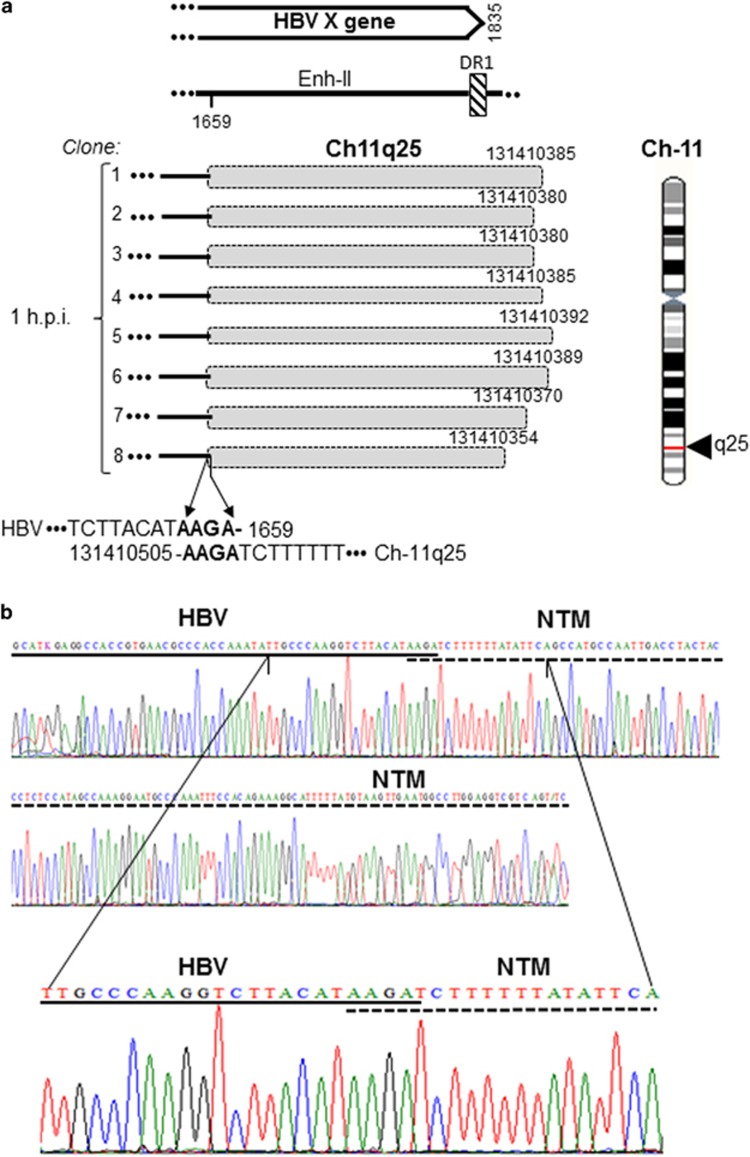
HBV integration with the neurotrimin (NTM) gene in HepaRG cells after 1-h exposure to NL02.C inoculum. (**a**) Schematic presentation of integration of HBx sequence (continuous lines) with NTM (shaded boxes) detected in eight separate clones (1–8). All clones displayed the same four-bp overlapping homolgous virus–host junction. Locations of the junction in Ch11q25 and in relation to the HBx sequence are shown. (**b**) Sequencing electropherograms depict in detail the nucleotides forming the OHJ. HBV and NTM sequences are marked by continuous and dashed lines, respectively. Enh-II, HBV enhancer II; DR1, direct repeat 1.

**Figure 3:**
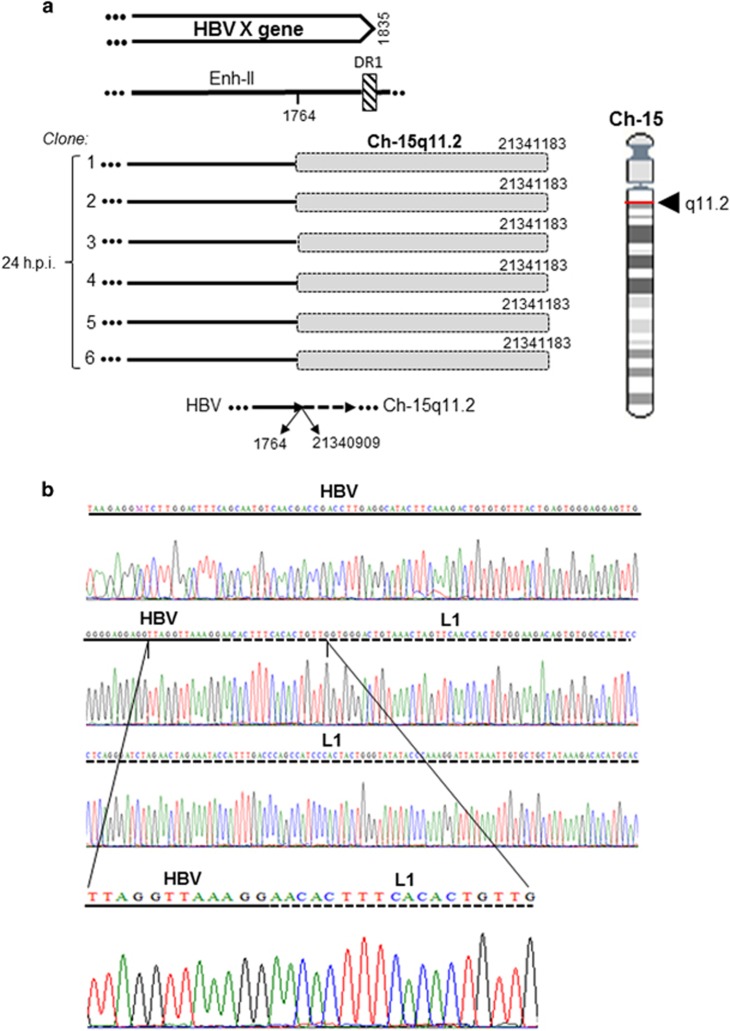
HBV DNA integration with LINE1 (L1) after 24-h exposure to HBV NL02.C. (**a**) Schemes showing HBx (continuous lines) integrated with LINE1 (shaded boxes) found in six independent clones (1–6). All clones demonstrated the same sequence of the head- to-tail junction. Location of the junction in Ch15 at q11.2 and in relation to HBx sequence are shown. (**b**) Electropherograms detailing the breaking point between HBV (continuous line) and LINE1 (dashed line). See the legend to Figure 2 for more details.

The formation of the earliest integration sites or VEIS was examined after HepaRG exposure to HBV N01.A or N02.C (Figure 1a). The first junctions were apparent after 1 h exposure. HBV N01.A produced multiple single site hits, whereas HBV N02.C formed a junction with the single site identified in all (8/8) clones caring integrations at this time point (Table 2). The site was within neurotrimin gene at chromosome (Ch) 11q25. It formed four-base pair (bp) overlapping homologous junction and had AAGA sequence created by nucleotides (nts) 1656–1659 of HBx and nts 131 410 505–131 410 502 of Ch11 (Figure 2 and Table 2). After 24-h exposure, HBV N02.C formed a junction with retrotransposon LINE1 located at Ch15q11.2 apparent in six clones. This was the head-to-tail join (HTJ) of HBx at nt 1764 with nt 21 340 909 of Ch15q11.2 (Figure 3). The VEIS involving LINE1 was also detected after 24-h exposure to NL01.A, but the LINE1 sequence engaged was located at Ch8p23.1 (Table 2). HBV NL01.A after 3 and 24-h exposure, and also at 3 d.p.i., formed a junction with the FLRT2 gene which the C-terminal sequence was joined with another retrotransposon, long-interspersed nuclear element-2 (LINE2 or L2) (Supplementary Figure 2). Interestingly, although these three integrations occurred at different time post infection, all formed HTJ with FLRT2 that was fused with LINE2 and all were located on Ch14q31.3.

### The early HBV–host DNA integrations

**Figure 4:**
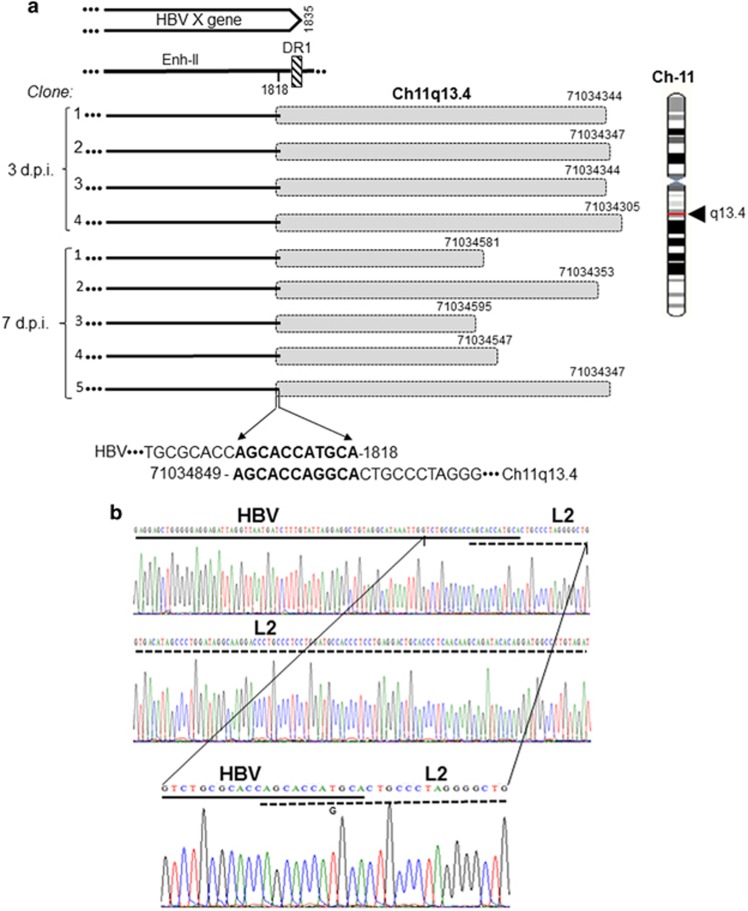
HBV integration with transposon LINE2 (L2) at 3 and 7 days post infection of HepaRG cells with NL01.A. (**a**) Integration of HBx (continuous lines) with LINE2 (shaded boxes) detected in four clones (1–4) obtained after 3 d.p.i. and in five clones (1–5) acquired at 7 d.p.i. (1–5). Different lengths of the LINE2 sequences identified, details on the HBV and LINE2 nucleotides forming the 11-bp homologous junction, except a singular nucleotide mismatch (T versus G), and location of the junction in relation to the HBx sequence and Ch11q13.4 are presented. (**b**) Electropherograms showing details on the contributions of HBV (continuous line) and LINE2 (dashed line) nucleotides to formation of the OHJ.

The EIS were defined as those forming junctions at 3 and 7 d.p.i. Thus, infection with HBV NL01.A produced at both time points integration with LINE2 evident in nine clones (Table 2). HBV-LINE2 fusion was of overlapping homologous junction type with the sequence AGCACCATGCA created by nts 1808–1818 of HBx and nts 71 034 849–71 034 839 of Ch11q13.4 (Figure 4). Also, a singular junction of HBV NL01.A with human satellite II DNA (HSAT-II) was identified at 3 d.p.i (Table 2). Regarding NL02.C, virus junctions with LINE1 were identified at 3 d.p.i. in two different locations on Ch8p23.1 (Table 2). NL02.C did not generate detectable integration signal at 7 d.p.i. (Figure 1a).

### Characteristics of the late HBV–host genome fusions

**Figure 5:**
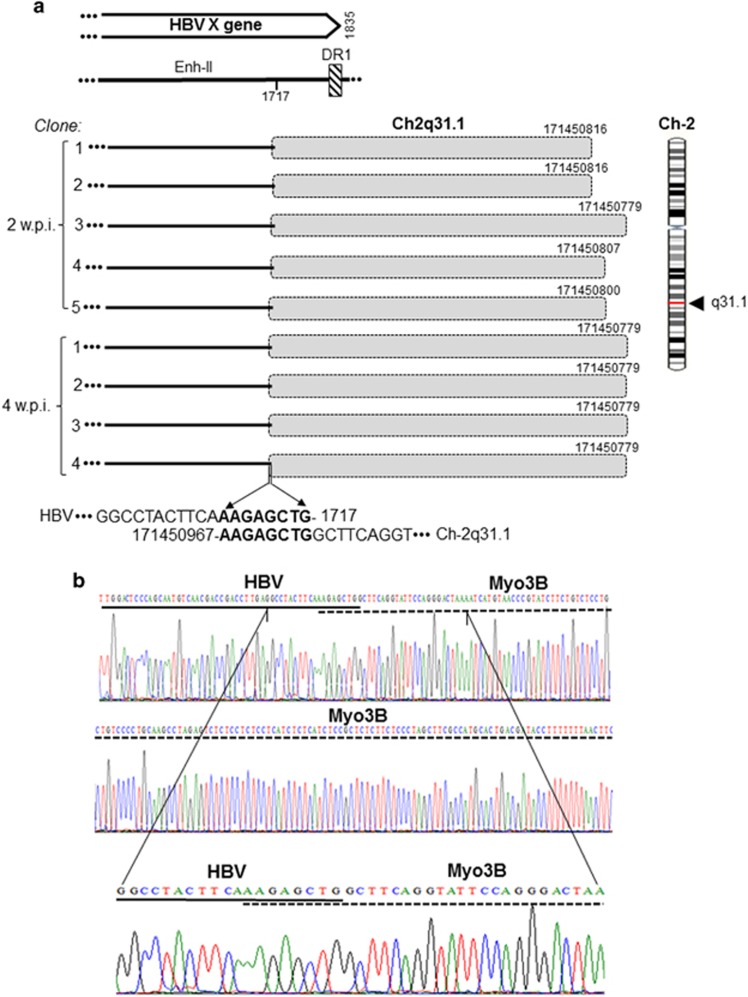
HBV integration with myosin III B (Myo3B) encoding gene in HepaRG cells at 2 and 4 weeks post infection with NL01.A. (**a**) Schematic presentation of integration of HBx sequence (continuous lines) with Myo3B (shaded boxes) detected in five (1–5) and four (1–4) clones obtained at 2 and 4 w.p.i., respectively. Different lengths of the Myo3 sequences, HBV and Myo3B nucleotides forming the eight-bp OHJ, and location of the integration in Ch11q13.1 and within the HBx sequence are shown. (**b**) Electropherograms detailing nucleotides forming the junction in relation to sequences of HBV (continuous line) and Myo3B (dashed line).

**Figure 6:**
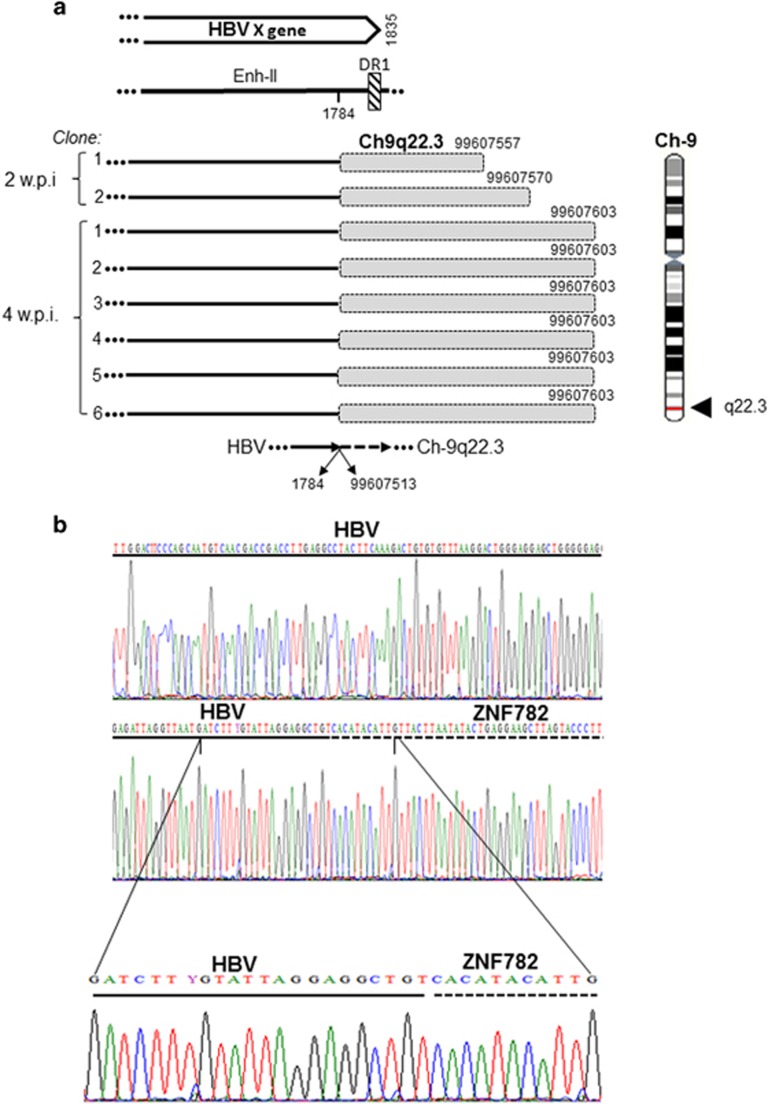
HBV junction with zinc finger protein 782 (ZNF782) encoding gene in HepaRG cells at 2 and 4 weeks post infection with NL01.A. (**a**) Integration of HBx sequence (continuous lines) with ZNF782 gene (shaded boxes) identified in two clones (1–2) obtained at 2 w.p.i. and in six clones (1–6) obtained at four w.p.i. Lengths of the ZNF782 sequences identified, nucleotides of the HBV and ZNF782 HTJ, and location of the junction in the HBx sequence and Ch11q25 are shown. (**b**) Electropherograms detailing the junction nucleotides in relation to the HBV (continuous line) and the ZNF782 (dashed line) sequences.

**Figure 7:**
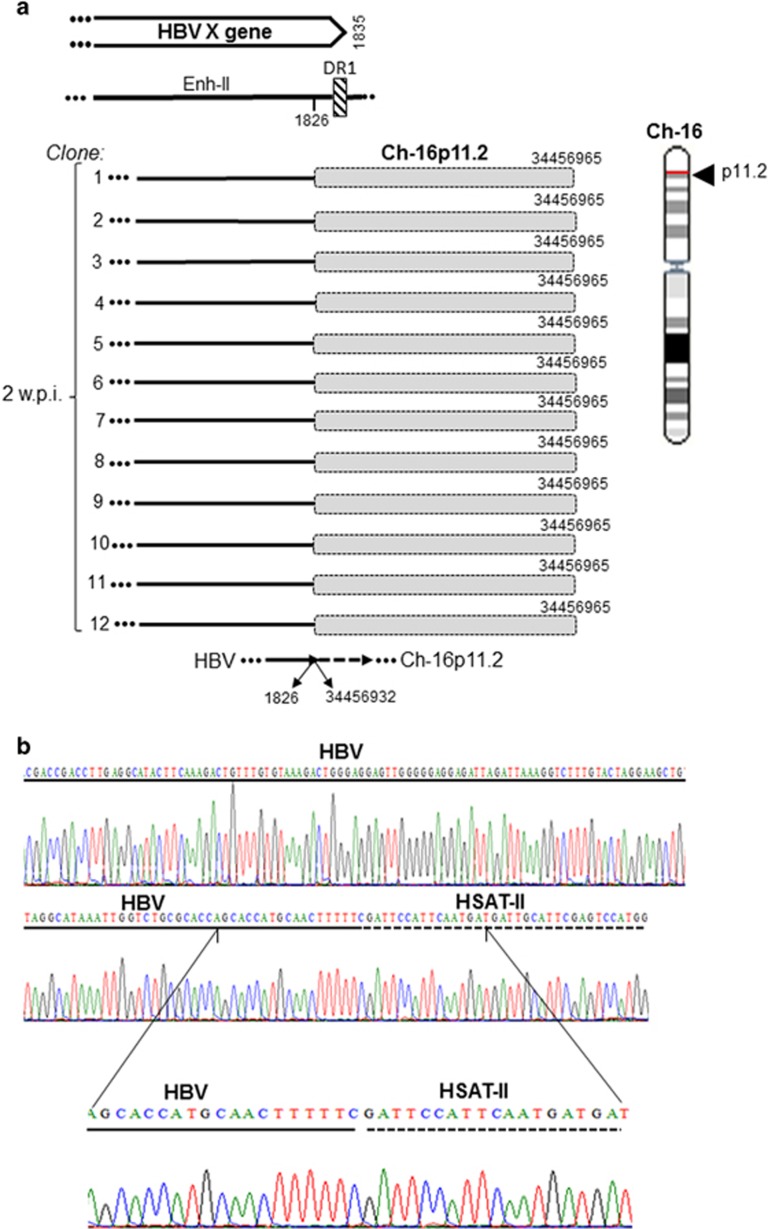
HBV DNA integration with retrotransposon human satellite DNA II (HSAT-II) DNA at 2 weeks after infection with NL03.E. (**a**) Schematic presentation of HBx (continuous lines) integrated with HSAT-II (shaded boxes) identified in 12 independent clones (1–12). All clones demonstrated the same HTJ sequence and locations within Ch16p11.2. (**b**) Electropherograms show in detail nucleotides forming the breaking point between HBV (continuous line) and HSAT-II (dashed line).

From 2 w.p.i. onward, HBV was found integrated at several sites (Table 2). One of the most prominent NEIS at 2 and 4 w.p.i. with HBV NL01.A was myosin III B at Ch2q31.1. This junction was the eight- bp overlapping homologous junction with sequence AAGAGCTG created by overlapping nts 1770–1717 of HBx and nts 171 450 967–171 450 960 of Ch2q31.1, and was confirmed in nine clones (Figure 5). Another prominent site was at gene encoding zinc finger protein 782 (ZNF782) on Ch9q22.3 forming the HTJ-type bond between HBx of HBV NL01.A at nt 1784 and nt 99 607 513 of Ch9q22.3 (Figure 6). Infection with NL03.E led to formation of yet another prominent junction with a tandemly repeating non-coding DNA, HSAT-II, located on Ch16p11.2. This HTJ was formed by HBx nt 1826 and nt 34 456 932 of Ch16p11.2 and was detected in 12 clones at 2 w.p.i. (Figure 7). As mentioned, a single hit at HSAT-II on Ch10q11.21 was also identified as an EIS after infection with NL01.A (Table 2).

In addition, there were several NEIS detected in singular clones, including: eEF1AP14 on Ch1q31.3 (Table 2), RNU7-147B on Ch2q33.1 (Table 2), ZBED3 on Ch5q13.3 (Supplementary Figure 3A), AC144568 on Ch8p23.1 (Table 2), and TET1 on Ch10q21.3 (Supplementary Figure 3B). All of them were HTJ with HBx.

### WHV–host DNA integrations

Three woodchucks showed WHV-specific signals after invPCR/NAH in liver biopsies collected at 1 or 3 h.p.i. (Figure 1b). These were integrations of the X gene (WHx) or preS region (WHpS), as verified by clonal sequencing. Several VEIS were identified (Table 3). The most prominent was that detected in Lbx-2 from Cw4/M, but the gene could not be assigned due to limited woodchuck sequence recognition. In two clones, WHx integrated at AAK1 gene on Ch2q14, based on >80% homology with human sequence (Table 3). Other VEIS were located at MAML2, KIA1117, PHACTR3 and LPIN3 genes. All of them formed HTJ with WHpS or WHx. Junctions were also identified in Lbx-3 samples acquired at 6 w.p.i. However, except for the HDAC-9 gene, their human equivalents could not be identified (Table 3). Identification of another site, NLRC5, was based on compatibility with the mouse genome. WHV integrations with MAML2, PHACTR3 and NLRC5 are shown in Supplementary Figure 4. Overall, this investigation confirmed that WHV, similar to HBV, integrates into host genome almost immediately after infection.

In addition, multiple virus–virus junctions were identified after infection with HBV or WHV (Tables 2 and 3). Although they were not the subject of this study, their existence at very early stages was consistent with detection of virus–host integrations and may suggest that similar mechanisms underlined their formation.

### Mapping of hepadnavirus X gene breaking points forming virus–host junctions

**Figure 8:**
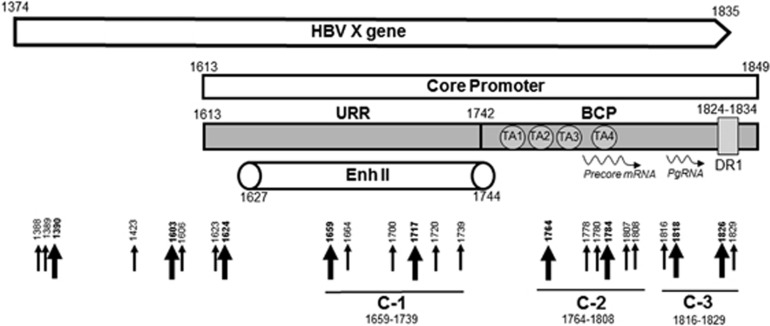
Schematic representation of the HBV X gene breaking points forming junctions with the human genomic sequences. Slim arrows identify breaking points which formed junctions detected in single clones, whereas bold arrows represent those identified in multiple clones. Four black circles depict HBV TATA elements. BCP, basal core promoter; DR, direct repeat region; Enh-II, HBV enhancer II region; Pg RNA, pre-genomic RNA; URR, upstream regulatory region. Numbers mark nucleotide positions according to HBV DNA GenBank X70185 sequence.

Most of the HBV–host junctions were formed by the HBx gene encompassing the core promoter (CP)/Enh-II region between nts 1613 and 1829 (Figure 8). Within this sequence, 75% (18/24) of all HBx breaking points were found, corresponding to 88.3% (68/77) of all hits. Further, 6/24 (25% 22 hits), classified as cluster-1 (C1) (Figure 8), were confined to Enh-II, whereas 10/24 (40.6% 42 hits) to basic core promoter (BCP). This clearly showed that the HBV BCP sequence was most prone to form junctions with host genome. The breaking points in the BCP were further divided into two clusters, C2 and C3 (Figure 8). The C2 spanned nts 1764–1808 that contained the TATA-like binding sequences (TA2–TA4) within nts 1758–1795, as well as pre-core mRNA initiation sites at nts 1788–1795. Six breaking points were identified in this region, including one with six hits at position 1764. The C3, encompassing nts 1816–1829, contains the HBV pre-genomic RNA initiation site at nt 1818.^29^ Four breaking points were found in this sequence, including one with nine hits at the initiation site.

Considering WHx, the junctions were predominantly located between nts 1810–1877 (10 hits at six sites), corresponding to the WHV BCP region. The preS breaking points were located within nts 3250–3308 (Table 3).

Distribution of hepadnavirus–host integration sites identified in this study across human chromosomes is shown in Supplementary Figure 5.

## Discussion

Integration of HBV DNA into hepatocyte genome has long been considered as the main contributor to liver oncogenesis and a potential determinant of virus persistence; however the timing of initiation of this process and the profile of virus insertional sites soon after infection remained unknown. In this study, we showed that HBV and its close relative WHV can integrate into host genome very soon after virus invasion, at the time of or shortly before viral replication became apparent in both infection models. Previously, supercoiled DNA of duck hepatitis B virus was identified as early as 6 h.p.i. and virus RNA transcripts at 12 h.p.i. in duckling livers by classical nucleic acid hybridization of relatively low sensitivity,^30^ whereas duck hepatitis B virus cccDNA from 9 h.p.i. in duck hepatocytes by PCR.^31^ The earliest HBV DNA integration was reported in hepatoma Huh7 cells at 8 d.p.i. by classical Southern blot analysis.^32^ In our study, the use of high sensitivity detection approaches and the cells from several minutes post exposure onwards shortened the time between the first contact with virus and identification of its replication and integration. This resembles the dynamics of HIV type 1 infection in cultured CD4+ T cells where viral reverse transcription occurred within 3 h.p.i. and virus integration at 8.5 h.p.i. as evaluated by quantitative PCR.^33^ The utilization of end-point PCR followed by NAH, which further enhanced sensitivity,^18, 19, 27^ was likely a reason behind our earlier detection of virus integrational signals.

To identify the initial sites of integration and their profiles across chromosomes, time-course experiments with authentic HBV were performed. The host sequences identified as the initial insertional sites were defined as those detected between 1 and 24 h after contact with virus. In the case of HBV, there appeared to be two profiles of virus integration. Thus, although infection with HBV NL01.A yielded junctions with several genes detectable in singular clones (Table 2), HBV NL02.C produced fusions with only two sites robustly presented in multiple clones. One of these sites was neurotrimin (Figure 2) encoding a neural adhesion molecule also occurring in stem and hepatic stellate cells,^34^ whereas another retrotransposon LINE1 (Figure 3). As all experimental and analytical conditions were identical, the difference might be related to the virus alone. Notably, these two inocula carried different HBV genotypes, A and C. In liver biopsies obtained at 1 or 3 h.p.i. from woodchucks infected with the same virus, WHV formed junctions with multiple genes localized on different chromosomes. This provided valuable confirmation that authentic hepadnavirus under natural *in vivo* infection conditions integrates within the same time frame as in our *in vitro* model and suggested that the initial integration at multiple rather than singular sites is the prevailing mode of hepadnavirus insertion. Undoubtedly, further studies will be required to determine potential influence of virus genotype on its insertional profile and if distinct integration patterns may coincide with different infection outcomes, including HCC development. The woodchuck–WHV model is in an advantageous position to advance our understanding of this and related issues.

In the period between 3 and 7 d.p.i., the HBV insertional sites, designated as EIS, almost exclusively encompassed LINE1 and LINE2 sequences represented in multiple clones (Table 2 and Figure 4). In addition, singular clones carrying HBV junctions with HSAT-II or FLRT2-L2 were detected after infection with HBV NL01.A (Table 2). Interestingly, in this last case, although virus integrated with FLRT2 gene, FLRT2 was also fused with a LINE2 sequence. This FLRT2-L2 merge was located 124-nt upstream from the HBV-FLRT2 junction (Supplementary Figure 2). This was validated by detection of the same HBV-FLTR2-L2 sequence at three different time points, that is, 3 and 24 h.p.i. and 3 d.p.i. (Table 2 and Supplementary Figure 2). In this regard, complex fusions and gene rearrangements were previously reported in the proximity to HBV DNA inserts.^9, 35, 36^ The function of FLTR2 is not defined but it is implicated in embryogenesis, organogenesis, and fibrogenesis.^37^ Dysregulation of genes involved in these processes have been linked to cancer development. The makeup of the HBx-FLRT2-L2 chimera may suggest a role in liver oncogenesis.

In the late post infection phase, multiple integration sites, classified as NEIS, were uncovered. Among them, HSAT-II had most hits (Table 2 and Figure 7). HBV integration at HSAT-II has not been reported, although HBV fusion with a related HSATIII was described in a hepatoma cell line and in tissues from HCC patients.^38, 39^ Another NEIS with frequent hits was the myosin III B gene at Ch2q31.1 (Figure 5), which encodes one of the actin-activated class III ATPses also occurring in hepatocytes.^40^ Frequent hits were also found with the ZNF782 (Figure 6) that encodes a nuclear DNA-binding protein likely functioning as a transcriptional factor.^41^ In liver biopsies from woodchucks at 6 w.p.i., WHV DNA was integrated at several genes and these insertions were largely in singular clones (Table 3).

One of the potentially most important findings was that HBV from the earliest stages of infection can integrate with retrotransposable elements, such as LINE1 and LINE2, and also HSAT-II. Considering the total number of insertional hits, almost a half (45.3%) were junctures with one of these sequences. This is in general agreement with recent studies showing that many HBV insertions occur in or near repetitive non-coding sequences, such as LINE, short interspersed nuclear elements and Alu.^7, 8, 9, 42^ Our finding of HBV integration with LINE retrotransposons, especially with LINE1, is particularly interesting. Human LINE1 is recognized as a major endogenous mutagen, a crucial source of mutations in HCC, and a mobile genetic element able to relocate sequences to new loci across chromosomes.^11, 12, 43^ Recent studies also uncovered that HBx-LINE1 integrants occur in a significant number of HBV-positive HCC and demonstrated pro-oncogenic properties of chimeric RNA transcribed from HBx-LINE1 fusion.^9, 10, 44^ HBx-LINE1 transcripts were shown to activate oncogenic β-catenin/Wnt signaling, promote epithelial–mesenchymal transition of hepatocytes,^9^ and sequester hepatocellular miR-122 fostering tumorigenic changes and liver tissue injury.^10^ Considering these properties of LINE1 and pro-oncogenic attributes of HBx-LINE1 transcripts, the finding of HBx-LINE1 integration very early after contact with virus implies that some of the processes begin to operate almost immediately after infection. This may also suggest that the molecular cascade of pro-oncogenic events could persist in the cell affected and its progeny long after resolution of hepatitis and during clinically silent infection, such as in occult infection preceding development of HCC.^13, 14, 15, 18, 19, 45^

HBx also formed junctions with retrotransposon LINE2 and another repetitive sequence, HSAT-II, but relevance of these interactions remain obscure. A role of HSAT-II derepression coinciding with overexpression of LINE1 was recently emphasized in the oncogenesis of epithelial cancers.^46, 47^ The postulated mechanism of upregulated HSAT-II expression in human carcinomas assumes a global dysregulation in DNA methylation, which is critical for the satellite repression.^46, 48^ Whether HBx-HASTII fusion may modify normally silenced HASTII has yet to be examined.

Our study also aimed at the delineation of integration breakpoints in HBV and WHV X gene, as this gene consistently demonstrated the greatest propensity to form virus–host junctures. Most of the HBV breakpoints detected in our study spanned within the DR regions and were categorized into three clusters (Figure 8). C1 was located within the Enh-II sequence, suggesting that the enhancer may hijack the transcriptional control of the fused host genes and involve them in liver pathogenic and tumorigenic perturbations. Also, as Enh-II can enhance expression of the downstream genes,^49^ the Enh-II fusions may modulate transcription of the genes not only directly linked but also those situated in the proximity of the junctions. C2 and C3 were located within nts 1764–1808 and 1816–1829, respectively, which span BCP (Figure 8). BCP has a central role in directing the transcriptional initiation of viral pre-core and pre-genomic mRNAs.^50^ Within the BCP region, there are four TATA box-like TA elements. TA1–TA3 are required for the optimal transcription of pre-core mRNA, whereas TA4 initiates downstream transcription of pre-genomic mRNA.^51^ HBx breakpoints within C2 and C3 may reduce or selectively abrogate transcription of viral RNA but they may also augment expression of the joined host sequences. Since virus–host integrations are common in HCC developing in the context of hepadnaviral occult infection, coinciding with very low rates of virus replication,^13, 18, 45^ this could be a mechanism uniting progression of occult infection with HCC development.

In this study, to investigate the initial hepadnavirus–host genome interactions, we applied: (1) two biologically relevant models of infection with authentic hepadnaviruses; (2) consecutive sampling of infected cells or livers beginning from very early time points after contact with pathogenic virus; (3) highly sensitive and specific approaches to detect viral genome integration and (4) detailed sequence analysis of virus–host junctions. The results demonstrate that the earliest integration of HBV and WHV occurs almost immediately after exposure to infectious virus. The results also showed that although HBV and WHV can form initial junctions with numerous genes, there is a recognizable trend to create HBx fusions with retrotransposon sequences. This appears in agreement with the latest findings in HBV-associated HCC and is of interest in light of oncogenic properties of HBx-LINE1 chimeric transcripts.

The mechanism of almost immediate hepadnavirus DNA integration into the host’s genome after infection remains hypothetical. An intriguing possibility could be a role of the ribonuclease H domain of hepadnavirus DNA polymerase that displays at least some properties of HIV integrase.^52, 53^ As this enzyme activity occurs in intact virions,^54^ it might facilitate initial hepadnavirus DNA integration even prior to detectable virus replication. Another probable mechanism could be very early activation of DNA repair machinery of double-strand DNA breaks, which are considered to be the prominent targets of hepadnaviral integration,^55^ likely owing to virus induced oxidative stress.^56, 57^ A finding of significantly augmented DNA damage in HepaRG cells at the time of detection of initial HBV integration might be consistent with this possibility (Supplementary Figure 6). The underlying molecular machinery will require meticulous dissection.

In conclusion, our study uncovered that the formation of virus–host genomic fusions is the very early event in hepadnaviral infection and that HBV insertions at host sites credited for important roles in hepatic oncogenesis may take place almost immediately after infection. In view of this, HBV must be considered as an unequivocal human carcinogen and that contact with this virus, including minuscule amounts causing primary occult infection,^16, 18, 58^ has to be utterly avoided.

## Materials and methods

### HepaRG cell culture and HBV infection

Undifferentiated HepaRG cells (a gift from Dr Camille Sureau, INTS, Paris, France and purchased from Invitrogen, Burlington, ON, Canada) tested for mycoplasma were cultivated in Williams E medium with supplements, as reported.^22^ For differentiation, 2% DMSO (Sigma-Aldrich, Oakville, ON, Canada) was added and cells cultured for 2 weeks. For infection, randomly selected cell cultures (~1.2 × 10^6^ cells/well) were exposed in duplicate to authentic HBV (Table 1) at a multiplicity of infection of 20 (~2.4 × 10^7^ DNase-digestion-protected virions) in the presence of 4% polyethylene glycol-8000. For infection of 24 h or less, cells were incubated with virus at 37 °C for the indicated time (Figure 1a), washed, harvested with 0.25% trypsin (Gibco-BRL, Burlington, ON, Canada), washed, treated with DNase-trypsin-DNase to remove potentially attached HBV and its DNA,^59, 60^ and nucleic acids extracted.^19, 60^ For infections lasting longer than 24 h, inoculum was removed after 24 h, and cells were washed, cultivated for the time specified (Figure 1a) and treated as above. Uninfected cells and those exposed to NHP for 24 h and cultured as infected cells served as controls. For each time point tested, cells from at least two independent experiments with each HBV inoculum or NHP were blindly analyzed.

Plasma from three treatment-naive patients with CHB without co-morbidities served as HBV inocula (Table 1). Patients were negative for antibodies to hepatitis C virus and HIV type 1 RNA. Plasma from healthy volunteers served as controls. Samples were collected after study approval by the Institutional Health Research Ethic Authority and signing written informed consent.

### Woodchucks and WHV infection

Four, 2–3-year old, randomly selected, healthy woodchucks (*Marmota monax*) (three males and one female) were intravenously injected with 1.1 × 10^10^ virions of WHV/tm3 inoculum (GenBank AY334075), as reported.^24^ Prior WHV exposure was excluded by negative testing of sera and liver biopsies obtained before WHV injection (Figure 1b), as described.^18, 19, 60^ LBx-2 were collected at 1 h.p.i. from two animals and at 3 h.p.i. from the remaining two (Figure 1b). LBx-3 were acquired 6 weeks thereafter from four animals. All woodchucks became serum WHV DNA-positive, virus surface antigen reactive at 14 d.p.i. The antigen cleared at 6 w.p.i. in three animals and at 8 w.p.i. in Cw1/M. The WHV infection status was known prior to this investigation. The animals were a part of the previous study determining expression of the intrahepatic, immune response-affiliated genes immediately after infection and during pre-acute and acute phases of WHV hepatitis.^24^ They were housed in the Woodchuck Viral Hepatitis Research Facility at Memorial University, St John’s, NL, Canada. All animal work was approved by the Institutional Committee on Animal Bioethics and Care.

### Detection of HBV and WHV genomes and replication

DNA was isolated as reported.^19, 60^ RNA was extracted with Trizol (Invitrogen), treated with DNase (Sigma-Aldrich) and reverse transcribed to cDNA.^19, 60, 61^ HBV and WHV DNA were assayed by direct and, if negative, nested PCR/NAH assays using primers and conditions described before.^18, 19, 60^ For HBV or WHV cccDNA detection assays previously reported were applied (sensitivity ~10^2^ copies/ml).^18, 62^ HBV or WHV RNA was detected by RT-PCR/NAH (sensitivity <10 copies/ml).^18, 19, 59, 61^ Viral DNA or RNA were also quantified by virus-specific real-time PCR or RT-PCR assays.^18, 61^ Mock extractions and nucleic acid preparations from livers of HBV- or WHV-positive carriers and uninfected HepaRG or normal woodchuck livers served as controls.^18, 19, 60^ NAH analysis was routinely conducted to verify the specificity of amplification signals, the validity of controls, and to enhance sensitivity of virus detection.

### Detection of hepadnavirus–host genome junctions

HBV–host junctions were identified by invPCR using conditions reported with modifications.^28^ Hence, HBx-specific primers were redesigned to amplify all HBV genotypes by nested PCR. The primers for direct PCR were: forward (F1) 5′-TTCGCTTCACCTCTGCACGT (1585–1604) and reverse (R1) 5′-5′-AAAGGACGTCCCKCGHAG (1405–1422); and for nested PCR were: forward (F2) 5′-GTYGCATGGARACCACCGTGA (1603–1623) and reverse (R2) 5′-CACARCCTAGCAGCCATGG (1372–1390) (nt positions according to GenBank X72702). InvPCR, products were probed by NAH to verify the presence of virus sequence. Confirmed products were digested with *Nco*-I and 10-fold serially diluted digests circularized with T4 DNA ligase, and then linearized.^28^ The possibility of self-ligated virus double-stranded DNA was excluded by digestion with *Sph*-I. Controls included DNA extracted from uninfected HepaRG cells, subjected or not to restriction enzyme digestion and ligation prior to invPCR/NAH, cells exposed to NHP instead of HBV, and cells exposed to HBV and immediately (time 0) subjected to all procedural steps. None of the controls showed detectable signals after invPCR/NAH. Also, DNA from 400 μl of plasma used as HBV or WHV inocula, processed as cellular DNA, were analyzed and no host sequences were detected.

For identification of WHV–host junctions, invPCR with primers specific for the WHV X and preS genomic regions was applied, as reported.^18^ DNA from Lbx-1, processed in parallel with Lbx-2 and Lbx-3 samples, served as negative controls. The presence of WHV signals was verified by NAH.^18, 19, 60^

### Sequence analysis

HBV sequence coordinates were mapped using BioEdit (Ibis Biosciences, Carlsbad, CA, USA) and sequences from GenBank as references: X70185 (genotype A), AB033556 (C), and X75657 (E). Human DNA sequences were identified and analyzed with NCBI BLAST (National Center for Biotechnology Information, Bethesda, MD, USA) and mapped for coordinates with BLAST genome browser (University of California at Sacramento) using human GRch37/hg19 sequence. WHV DNAs were mapped to the full-length WHV/tm3 sequence (GenBank AY334075) using BioEdit. Identification of retrotransposable sequences was done using Dfam browser for repetitive elements and SINEBase for short interspersed sequences.^63, 64^

## Figures and Tables

**Table 1:** Virological characteristics of patients with chronic hepatitis B who provided HBV inocula

*Inoculum*	*Patient age (years)/sex*	*HBV DNA(IU/ml)*^a^	*HBV genotype*^b^	*HBsAg*^c^	*HBeAg*^c^	*Anti-HBe*^c^	*Anti-HBc*^c^	*HIV RNA*^d^	*Anti-HCV*^e^
NL01	58/M	2.7 × 10^7^	A	pos	pos	neg	pos	neg	neg
NL02	35/F	1.1 × 10^8^	C	pos	n.a.	n.a.	pos	neg	neg
NL03	33/M	5.8 × 10^8^	E	pos	pos	n.a.	pos	neg	neg

Abbreviations: anti-HBc, antibodies to HBV core antigen; anti-HBe, antibodies to HBeAg; anti-HCV, antibodies to hepatitis C virus; F, female; HBeAg, HBV e antigen; HBsAg, HBV surface antigen; IU, international units; M, Male; n.a., not available.

a Determined by COBAS Ampliprep/TaqMan HBV Test (sensitivity 20 IU/ml) Roche Diagnostics, Laval, Quebec, Canada.

b Assayed by in-house PCR with HBV S gene-specific primers (forward primer: 5′-GCCTCATTTTGTGGGTCACCATA-3' and reverse primer: 5′-ATAACTGAAAGCCAAACAGTGGG-3') and bidirectional sequencing of the resulting 1115-bp amplicons.

c Determined by ARCHITECT immunoassays from Abbott Diagnostics, Mississauga, Ontario, Canada.

d Tested by Abbott Real Time HIV-1 assay m2000 (sensitivity 75–10^7^ virus copies/ml), Abbot Diagnostics, Mississauga, Ontario, Canada.

e Determined by COBAS Amplicor HCV v2.0 (sensitivity 50–60 IU/ml), Roche Diagnostics, Laval, Quebec, Canada.

**Table 2:** HBV-human genome junctions identified after *de novo* infection

*HBV inoculum/ time post infection*	*Total of virus–host junctions detected (no)*	*Integrated HBV sequence (nt. position)*	*Host sequence (bp length)*	*Chromosome number*	*Sequence locus*	*Host sequence (nt. position)*	*Gene*	*Total of virus–virus junctions detected (no)*
*NL01.A*								
1 h	4	1720–1603	17	1	q21.1	420–437	ANP32E	0
		1639–1829	18	7	q22.1	98457063–98457081	S3A-26	
		1647–1720	40	10	q21.2	111339527–111339567	ANK3	
		1662–1603	48	13	q33.1	102521074 102521122	FGF14	
3 h	2	1645–1700	27	14	q32.3	54–27	UI	14
		1603–1624	204	14	q31.3	86038392–86038596	FLRT2/L2	
24 h	4	1246–1390	20	1	p21.3	97732036–97732056	DPYD	3
		1370–1423	16	7	q36.1	60109931–60109947	RNY-1	
		1603–1623	141	8	p23.1	7874512–7874653	L1	
		1603–1624	204	14	q31.3	86038392–86038596	FLRT2/L2	
3 d	6	1371–1390	244	10	q11.21	42599732–42599976	HSAT-II	3
		1647–1818	505	11	q13.4	71034849–71034344	L2	
		1647–1818	502	11	q13.4	71034849–71034347	L2	
		1647–1818	505	11	q13.4	71034849–71034344	L2	
		1647–1818	544	11	q13.4	71034849–71034305	L2	
		1603–1624	204	14	q31.3	86038392–86038596	FLRT2/L2	
1 w	5	1647–1818	268	11	q13.4	71034849–71034581	L2	12
		1647–1818	496	11	q13.4	71034849–71034353	L2	
		1647–1818	254	11	q13.4	71034849–71034595	L2	
		1634–1818	302	11	q13.4	71034849–71034547	L2	
		1634–1818	502	11	q13.4	71034849–71034347	L2	
2 w	10	1603–1664	19	1	q31.3	194161891–194161910	eEF1AP14	7
		1623–1717	151	2	q31.1	171450967–171450816	Myo3B	
		1638–1717	151	2	q31.1	171450967–171450816	Myo3B	
		1645–1717	188	2	q31.1	171450967–171450779	Myo3B	
		1623–1717	160	2	q31.1	171450967–171450,807	Myo3B	
		1622–1717	167	2	q31.1	171450967–171450800	Myo3B	
		1638–1717	220	2	q33.1	2000084860–200085080	RNU7–147 P	
		1276–1389	59	2	q33.1	200085021–200085080	RNU7–147 P	
		1638–1784	44	9	q22.3	99607513–99607557	ZNF782	
		1642–1784	57	9	q22.3	99607513–99607570	ZNF782	
4 w	11	1634–1717	188	2	q31.1	171450967–171450779	Myo3B	5
		1633–1717	188	2	q31.1	171450967–171450779	Myo3B	
		1633–1717	188	2	q31.1	171450967–171450779	Myo3B	
		1634–1717	188	2	q31.1	171450967–171450779	Myo3B	
		1645–1784	90	9	q22.33	99607513–99607603	ZNF782	
		1645–1784	90	9	q22.33	99607513–99607603	ZNF782	
		1645–1784	90	9	q22.33	99607513–99607603	ZNF782	
		1645–1784	90	9	q22.33	99607513–99607603	ZNF782	
		1645–1784	90	9	q22.33	99607513–99607603	ZNF782	
		1645–1784	90	9	q22.33	99607513–99607603	ZNF782	
		1668–1808	276	10	q21.3	70434086–70434362	TET1	
7 w	3	1634–1826	32	2	q37.1	235357695–235357727	RPS20P12	7
		1643–1826	35	8	p23.1	96050–96085	AC144568	
		1645–1739	60	10	p11.23	28765451–28765521	WAC-AS1	
*NL02.C*								
1 h	8	1616–1659	120	11	q25	131410505–131410385	NTM	11
		1606–1659	125	11	q25	131410505–131410380	NTM	
		1606–1659	125	11	q25	131410505–131410380	NTM	
		1624–1659	120	11	q25	131410505–131410385	NTM	
		1606–1659	113	11	q25	131410505–131410392	NTM	
		1606–1659	116	11	q25	131410505–131410389	NTM	
		1606–1659	135	11	q25	131410505–131410370	NTM	
		1606–1659	151	11	q25	131410505–131410354	NTM	
24 h	6	1645–1764	274	15	q11.2	21340909–21341183	L1	0
		1645–1764	274	15	q11.2	21340909–21341183	L1	
		1645–1764	274	15	q11.2	21340909–21341183	L1	
		1645–1764	274	15	q11.2	21340909–21341183	L1	
		1645–1764	274	15	q11.2	21340909–21341183	L1	
		1645–1764	274	15	q11.2	21340909–21341183	L1	
3 d	2	1625–1606	215	8	p23.1	7441825–7442040	L1	4
		1624–1603	189	8	p23.1	7150697–7150886	L1	
2 w	2	1716–1780	79	1	p2.1	148552682–148552761	NBPF25P	2
		1390–1388	208	5	q13.3	76373689–76373809	ZBED3	
*NL03.E*								
7 d	1	1641–1778	271	10	q21.3	146–417	S3A-37	2
2 w	12	1647–1826	33	16	p11.2	34456932–34456965	HSAT-II	1
								
		1659–1826	35	16	p11.2	34456932–34456967	HSAT-II	
		1646–1826	39	16	p11.2	34456932–34456971	HSAT-II	
		1647–1816	34	16	p11.2	34456932–34456966	HSAT-II	
		1647–1826	34	16	p11.2	34456932–34456966	HSAT-II	
		1647–1826	35	16	p11.2	34456932–34456967	HSAT-II	
		1647–1826	34	16	p11.2	34456932–34456966	HSAT-II	
		1647–1826	34	16	p11.2	34456932–34456966	HSAT-II	
		1647–1826	34	16	p11.2	34456932–34456966	HSAT-II	
		1647–1826	34	16	p11.2	34456932–34456966	HSAT-II	
		1647–1826	34	16	p11.2	34456932–3445696	HSAT-II	
		1647–1826	34	16	p11.2	34456932–34456966	HSAT-II	

Abbreviations: AC144568, uncharacterized RNA gene; ANK3, ankyrin3; ANP32E, acidic (leucine rich) nuclear phosphoprotein 32 family, member E; DPYD, dihydropyrimidine dehydrogenase; eEF1AP14, eukaryotic elongation factor 1 ribosomal protein; FGF14, fibroblast growth factor 14; FLRT2, fibronectin leucine rich transmembrane protein; HSAT-II, human satellite II DNA; L1, long-interspersed nuclear element-1; L2, long-interspersed nuclear element-2; Myo3B, myosin III B; NTM, neurotrimin; NBPF25P, neuroblastma breakpoint family member 25 pseudogene; RNU7-147P, RNA U7 small nuclear 147 pseudogene; RNY-1, Ro-associated Y pseudogene 1; RPS20P12, ribosomal protein S20 pseudogene 12; S3A-26, ribosomal protein S3A pseudogene 26; S3A-37, ribosomal protein S3A pseudogene 37; TET1, ten-eleven translocation 1 gene; UI, unidentified sequence; WAC-AS1, WAC antisense RNA gene; ZBED3, zinc finger, BED-type containing 3; ZNF782, zinc finger protein 782.

**Table 3:** WHV–woodchuck genome junctions identified in liver biopsies after *de novo* infection

*Animal/ liver biopsy number*	*Total of virus–host junctions detected (no)*	*WHV sequence (nt. position)*	*Host sequence (bp length)*	*Chromosome number*^a^	*Sequence locus*^a^	*Host sequence (nt. position)*	*Gene*^a^	*Total of virus–virus junctions detected (no)*
Cw1/M LBx2-1 h.p.i.	3	PS3200–3256	33	11	q21	95720503–95720470	MAML2	3
		X1853–1876	16	UI	UI	UI	UI	
		PS2785–2934	36	15	q25	84783482–84783518	EFTUD1P1	
Cw2/F LBx2-1 h.p.i.	5	X1913–1853	48	2	p14	69870009–69870057	AAK1	12
		X1913–1853	118	2	p14	69784039–69784157	AAK1	
		X1913–1853	19	6	q14.1	83777393–8377412	KIAA1117	
		X1910–1853	20	20	q12	39989149–39989169	LPIN3	
		PS3030–3009	17	20	q13.2	178193731–178193748	PHACTR3	
Cw4/M LBx2–3 h.p.i.	3	PS3250–3308	75	UI	UI	UI	UI	4
		PS3251–3308	68	UI	UI	UI	UI	
		PS3251–3308	70	UI	UI	UI	UI	
Cw1/M LBx3–6 w.p.i.	2	X1855–1876	16	UI	UI	UI	UI	0
		X1854–1877	18	16^b^	q13^b^	2650–2632	NLRC5^b^	0
Cw2/F LBx3–6 w.p.i.	2	X1623–1501	210	UI	UI	UI	UI	0
		X1548–1501	137	UI	UI	UI	UI	
Cw3/F Bx3–6 w.p.i.	1	X1772–1821	81	UI	UI	UI	UI	2
Cw4/M LBx3–6 w.p.i.	2	PS3250–3308	68	UI	UI	UI	UI	4
		X1651–1810	26	X	p21.1	18674290–18674316	HDAC-9	

Abbreviations: AAK1, AP-1 associated kinase; EFTUD1P1, elongation factor Tu GTP binding domain containing 1 pseudogene; HDAC-9, histone deacetylase-9; Kazusa cDNA 1117; KIAA1117; LPIN3, lipin-3; MAML2, mastermind-like 2; NLRC5, NLR family CARD domain containing 5; PHACTR3, phosphatase and actin regulator 3; UI, unidentified sequence.

a Based on homology with human genome.

b Based on homology with mouse genome.
