# Supplementary material for: Initial sites of hepadnavirus integration into host genome in human hepatocytes and in the woodchuck model of hepatitis B-associated hepatocellular carcinoma
Source: Oncogenesis. 2017 Apr 17;6(4):e317–. doi: 10.1038/oncsis.2017.22 (PMC5520499; doi:10.1038/oncsis.2017.22)
Supplement: Supplementary Information [file oncsis201722x1.pdf]

SUPPLEMENTARY INFORMATION

Initial sites of hepadnavirus integration into host genome in human hepatocytes and in the woodchuck model of hepatitis B-associated hepatocellular carcinoma

Ranjit Chauhan, Norma D. Churchill, Patricia M. Mulrooney-Cousins & Tomasz I. Michalak

Molecular Virology and Hepatology Research Group, Division of BioMedical Sciences, Faculty of Medicine, Health Sciences Centre, Memorial University, St. John's, NL A1B 3V6, Canada

Supplementary Figures:

SI-Fig. 1. Detection of HBV DNA signals indicating virus-host genome integrations by invPCR/NAH in HepaRG cells.

SI-Fig. 2. HBV DNA integration with FLRT2 gene containing sequence of retrotransposon LINE2 (L2) identified in HepaRG cells after 3-h and 24-h exposure and 3 days post-infection with HBV NL01.A inoculum.

SI-Fig. 3. HBV integrations detected in HepaRG cells at two or more weeks after infection with HBV NL01.A or HBV NL02.C.

SI-Fig. 4. Examples of WHV DNA integration detected in liver biopsies from two woodchucks infected with the same virus.

SI-Fig. 5. Distribution of the identified sites of hepadnavirus integration across the human genome.

SI-Fig. 6. Increased HepaRG cell DNA damage coincides with very early HBV DNA integration post-infection.

Supplementary Tables:

SI-Table 1. GenBank accession numbers for HBV-human junctions identified in this study.

SI-Table 2. GenBank accession numbers for WHV-woodchuck junctions identified in this study.

SI - Fig. 1

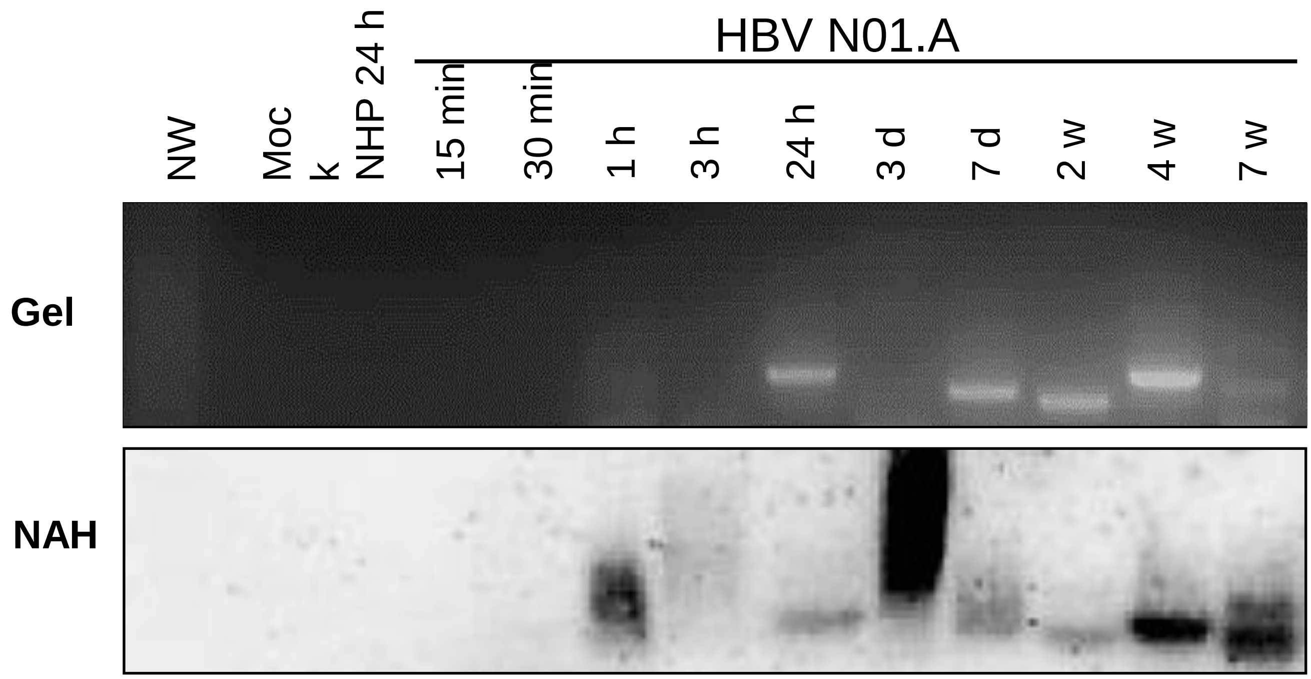

Detection of HBV DNA signals indicating virus-host genome integrations by nested invPCR/NAH in HepaRG cells. An example of detection of HBV DNA integration signals in HepaRG cells after exposure to HBV N01.A inoculum and collection of the cells after the time periods indicated (from one hour to 7 w.p.i.). Signals were detected by invPCR with HBV X gene-specific primers after gel electrophoresis (gel) and subsequent probing by hybridization with full-length recombinant HBV DNA (NAH). Only signals confirmed by NAH were further analyzed. Contamination and specificity controls: NW, water added instead of test DNA and amplified by a direct and then a nested invPCR; Mock, DNA from uninfected HepaRG treated exactly as DNA from infected cells; NHP24h, HepaRG cells exposed to normal human plasma for 24 h, processed as the cells exposed to HBV, and their DNA treated exactly as that from infected cells.

SI - Fig. 2

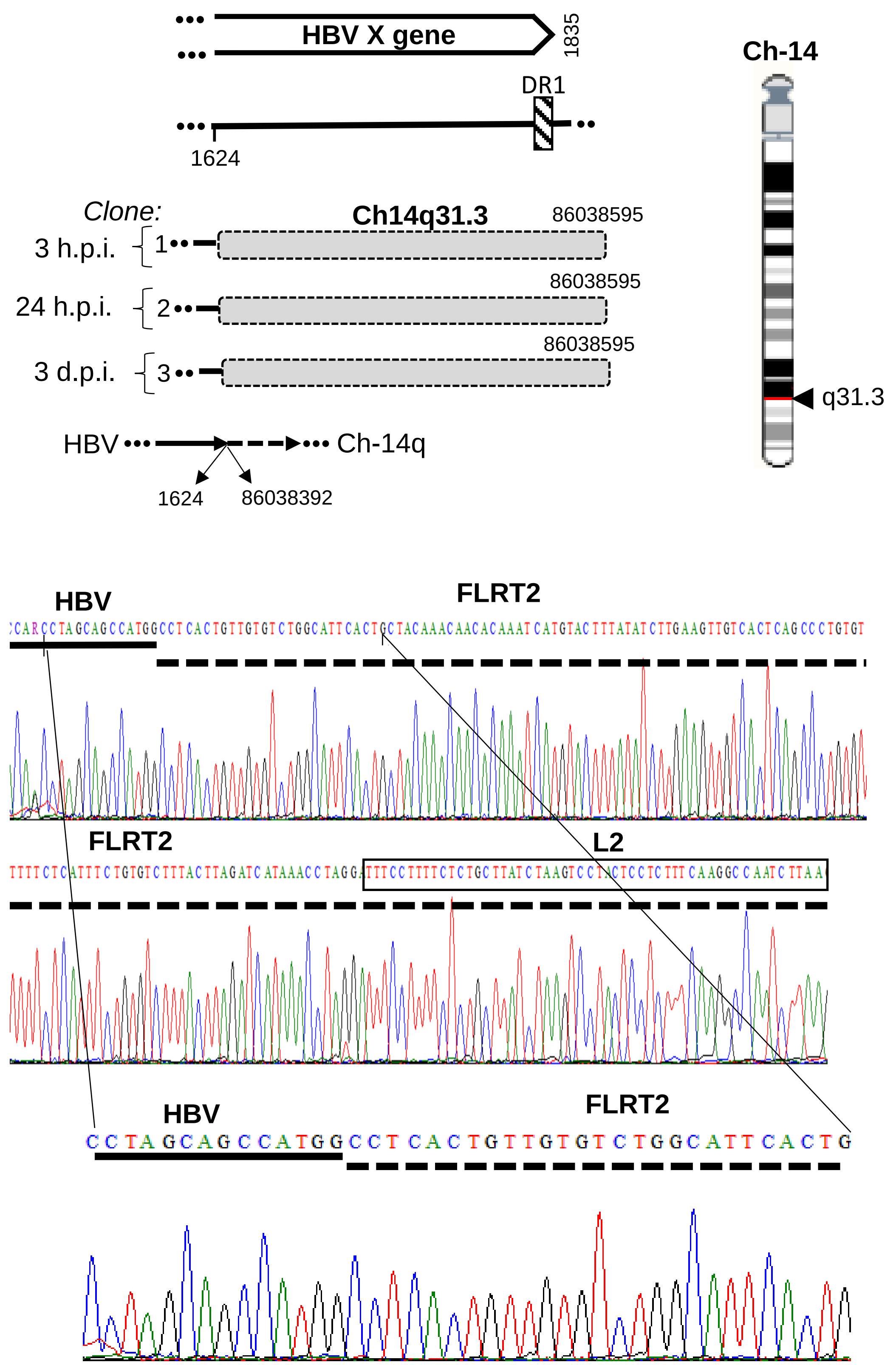

HBV DNA integration with FLRT2 gene containing sequence of retrotransposon LINE2 (L2) identified in HepaRG cells after 3-h and 24-h exposure and 3 days post-infection with NL01.A. HBV-FLRT2/L2 integrants displaying the same head-to-tail junction and fused LINE2 sequence were identified at all 3 time points. Electropherogram detailing HBV and FLRT2 join and its location in Ch14 are shown. HBV and FLRT2 sequences are underlined by continuous and dashed lines, respectively, while LINE2 sequence is denoted by a rectangle square.

SI - Fig. 3

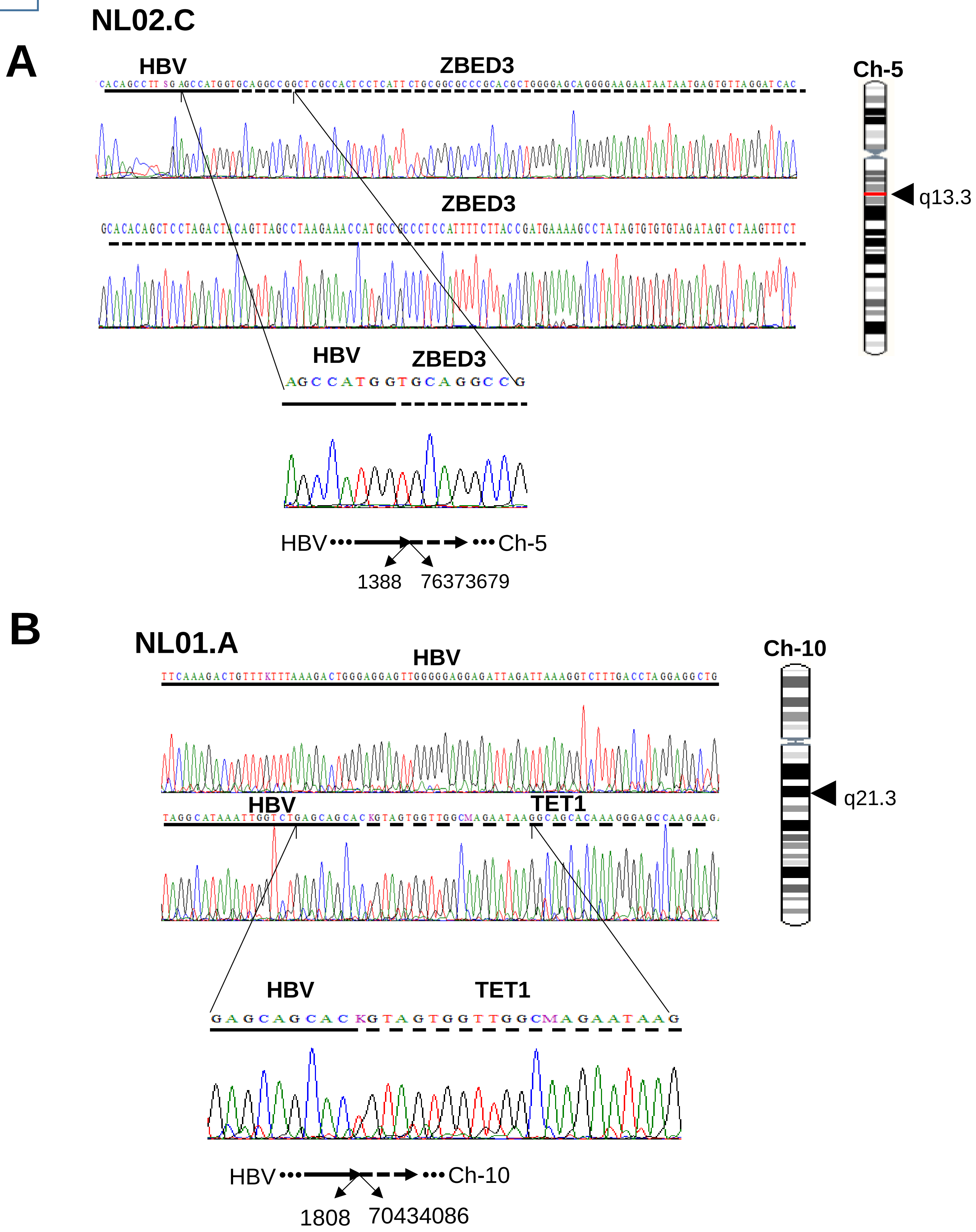

HBV integrations detected in HepaRG cells at two or more weeks after infection with HBV NL01.A or HBV NL02.C. (**A**) HBV DNA junction with zinc finger BED-type containing 3 gene (ZBED3) on Ch5q13.3 at 2 w.p.i. with NL02.C and (**B**) Integration of HBV with ten eleven translocation gene-1 (TET1) on Ch10q21.3 identified at 4 w.p.i. with NL01.A. Both HBV-host DNA fusions were of HTJ type. Location of the junctions in chromosomes and electropherograms detailing nucleotides at the breaking points are shown.

SI - Fig. 4

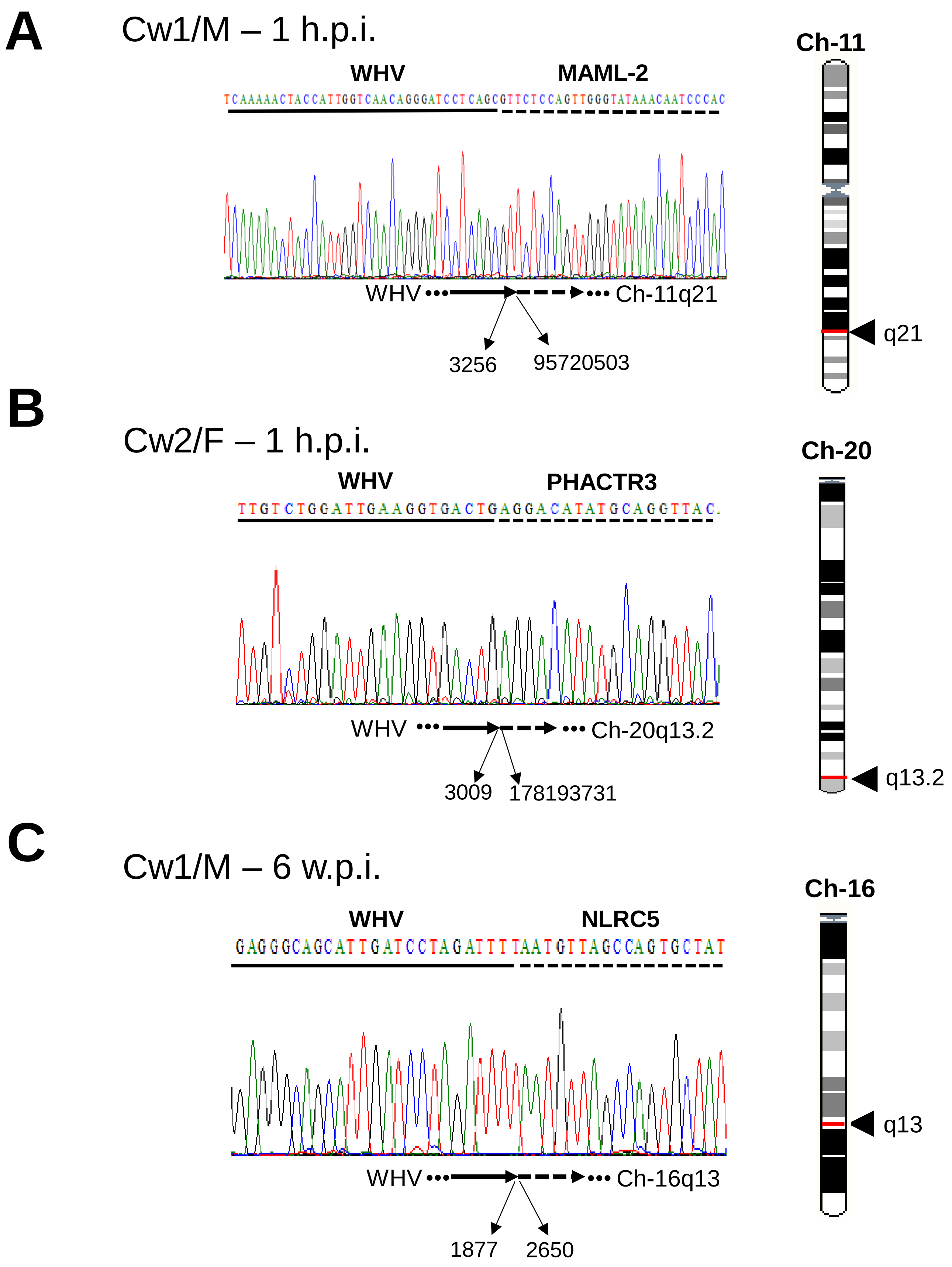

Examples of WHV DNA integration detected in liver biopsies from two woodchucks infected with the same virus.
(A) WHV DNA junction with mastermind like-2 gene2 (MAML2) in liver biopsy obtained from Cw1/M at one hour
(B) post-infection. (**B**) WHV DNA integration with sequence of phospahatase and actin regulator 3 (PHACTR3)
(C) in liver sample obtained at one hour post-infection from Cw2/F woodchuck. (**C**) WHV DNA integration with NLR
(D) family CARD domain containing 5 (NLRC5) in liver biopsy acquired from Cw1/M at 6 weeks post-infection.
(E) All three virus-host DNA fusions were of HTJ type.

SI - Fig. 5

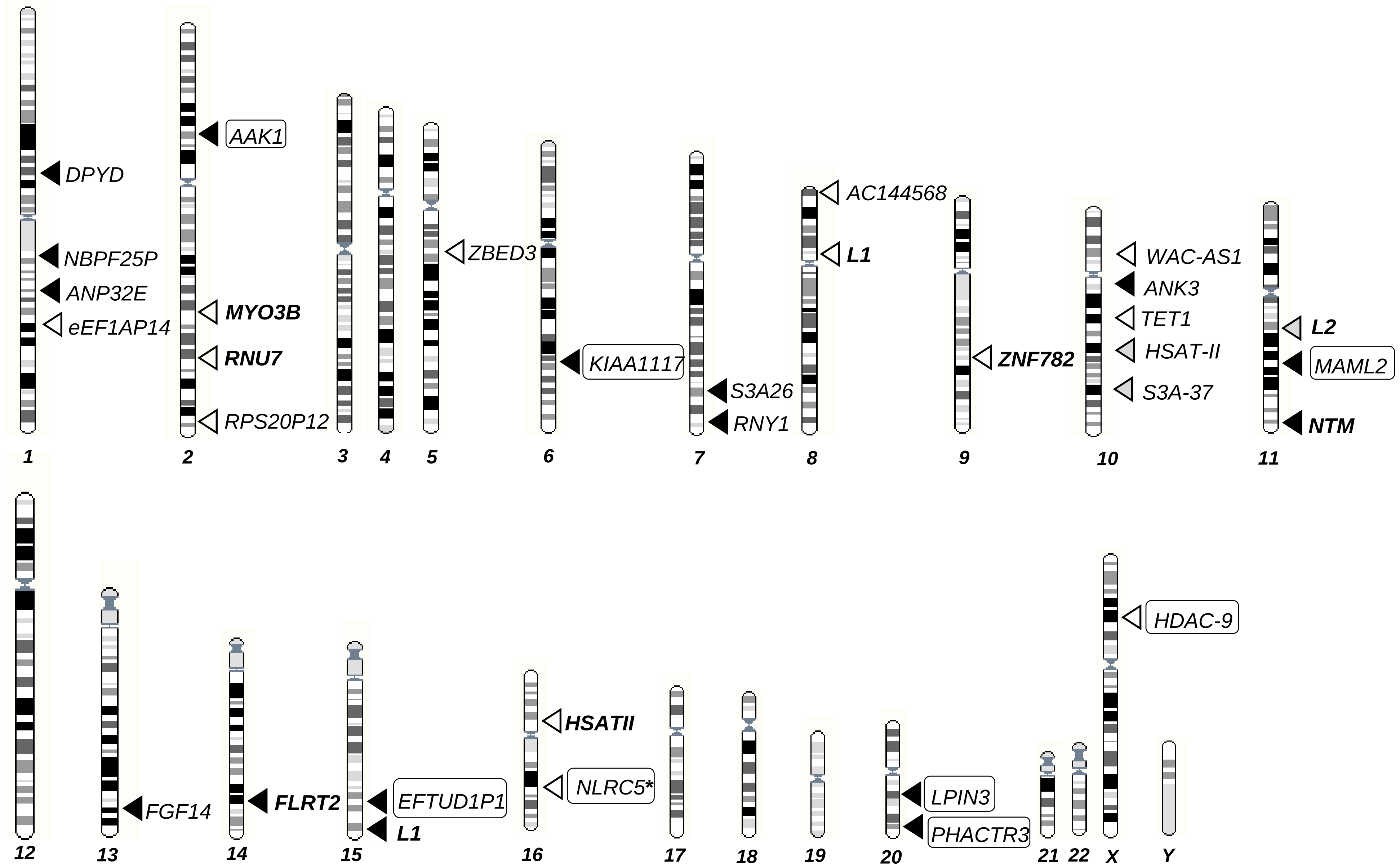

Distribution of the identified sites of hepadnavirus integration across the human genome. Locations of HBV and WHV integrations were imposed on the diagrams of human chromosomes. In the case of WHV-host junctions, the positions were assigned based on the site sequence compatibility with the human sequence, except NLRC5 (*) that was mapped based on its compatibility with mouse genome sequence. Black triangle (◀) indicates HBV very early integration site (VEIS; ≤24-h post-infection); gray triangle (◀) HBV early integration site (EIS; 3-7 days post-infection), open triangle (◁) HBV late integration sites (NEIS; ≥2 weeks post-infection), and the framed gene names mark woodchuck-WHV integration sites. The names in bold indicate the genes with multiple integration hits. Considering formation of the HBV-host junctions, Ch10 and Ch1 were most frequently targeted (5 and 4 different sites, respectively). Seven chromosomes showed one or two hits, and Ch2 three different integration sites. 9/23 chromosomes showed no evidence of HBV integration. The distribution of WHV integrants did not reveal preference for targeting any identifiable genomic site.

SI - Fig. 6

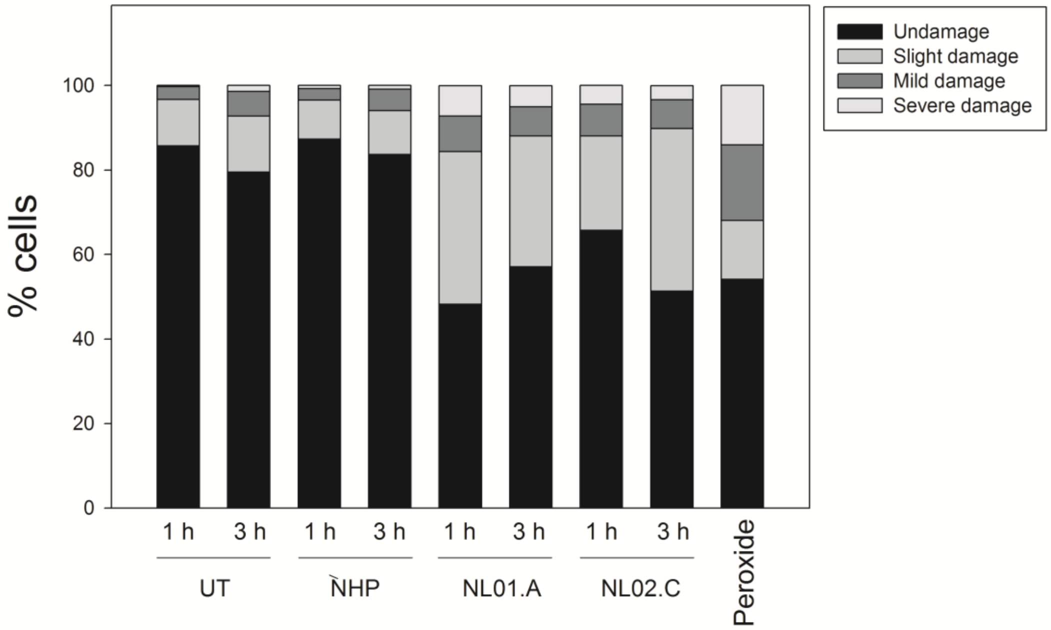

Increased HepaRG cell DNA damage coincides with very early HBV DNA integration post-infection. DNA damage was evaluated by the comet assay using the alkaline single cell gel protocol (Integrated Laboratory Systems, Research Triangle Park, NC). HepaRG cells were exposed to HBV NL01.A or HBV NL02.C, normal human plasma (NHP) or left untreated (UT; *i.e.,* in the presence of the same culture medium as that used for infection) for 1 or 3 h, as described in Materials and Methods. Cells exposed to 200 µM peroxide in the absence of HBV inocula or NHP served as DNA damage-positive controls. After alkaline electrophoresis, nuclei were stained with SYBR green (Invitrogen) and blindly analyzed under an epifluorescent microscope by two investigators. For each sample, at least 400 nuclei were examined. The length of the comet tail corresponding to the severity of DNA damage in each nucleus was classified into four categories: 0, no tail (undamaged); 1, short (slight damage); 2, moderate (mild damage) and 3, between moderate and maximum (severe damage). All evaluations were performed in duplicate after two separate cell treatments under the conditions described. DNA damage detected by the comet assay in the cells exposed to HBV was significantly greater (except those exposed to HBV NL02.C for 1 h) than in those exposed to NHP or kept UT (*P* 0.034-<0.001) and was comparable to the cells treated with peroxide for 1 h serving as a DNA damage-positive control (see Table below for significance). For comparison of the total percentages of cell nuclei exhibiting DNA damage (*i.e.,* slight, mild and severe), a one-way analysis of variance (ANOVA) with a post-hoc multiple range Tukey’s test was performed.

Exact P values for comparisons between the total percentages of comets detected in HepaRG cells exposed to native HBV NL01.A or HBV NL02.C, normal human plasma (NHP), left untreated (UT) or treated with peroxide as a DNA damage-positive control

	UT 1 h	***UT 3 h***	***NHP 1 h***	***NHP 3 h***	***NL01.A 1 h***	***NL01.A 3 h***	***NL02.C 1 h***	***NL02.C 3 h***	***Peroxide 1 h***
UT 1 h	X	ns	ns	ns	<0.001	0.017	ns	0.002	0.001
UT 3 h	ns	X	ns	ns	<0.001	0.024	ns	0.002	0.001
NHP 1 h	ns	ns	X	ns	<0.001	0.001	ns	<0.001	<0.001
NHP 3 h	ns	ns	ns	X	<0.001	0.034	ns	0.004	0.001
NL01.A 1 h	<0.001	<0.001	<0.001	<0.001	X	ns	0.001	ns	ns
NL01.A 3 h	0.017	0.024	0.001	0.034	ns	X	ns	ns	ns
NL02.C 1 h	ns	ns	ns	ns	0.001	ns	X	0.007	0.002
NL02.C 3 h	0.002	0.002	<0.001	0.004	ns	ns	0.007	X	ns
Peroxide 1 h	0.001	0.001	<0.001	0.001	ns	ns	0.002	ns	X

Analysis was performed using a one-way ANOVA with a post-hoc multiple range Tukey's test for significant values. *P* considered significant when $\leq$0.05. ns, not significant. Fields in grey, exposure to NL01.A for 1 and 3 h and to NL02.C for 3 h gave significantly greater total percentages of cells with comets than after exposure to NHP for 1 or 3 h or in cells left UT for the same period of time, which also were comparable to those detected after exposed to peroxide for 1 h.

SI-Table 1. GenBank accession numbers for HBV-human junctions identified in this study

HBV inoculum	Time post-infection	Gene	Accession number
**NL01.A**	1 h	ANP32E	KS372512
		S3A-26	KS372513
		ANK3	KS372514
		FGF14	KS372515
	3 h	UI	KS372516
		FLRT2/L2	KS372517
	24 h	DPYD	KS372518
		RNY-1	KS372519
		L1	KS372520
		FLRT2/L2	KS372521
	3 d	HSAT-II	KS372522
		L2	KS372523
		L2	KS372524
		L2	KS372525
		L2	KS372526
		FLRT2/L2	KS372527
	1 w	L2	KS372528
		L2	KS372529
		L2	KS372530
		L2	KS372531
		L2	KS372532
	2 w	eEF1AP14	KS372533
		Myo3B	KS372534
		Myo3B	KS372535
		Myo3B	KS372536
		Myo3B	KS372537
		Myo3B	KS372538
		RNU7-147P	KS372539
		RNU7-147P	KS372540
		ZNF782	KS372541
		ZNF782	KS372542
	4 w	Myo3B	KS372543
		Myo3B	KS372544
		Myo3B	KS372545
		Myo3B	KS372546
		ZNF782	KS372547
		ZNF782	KS372548
		ZNF782	KS372549
		ZNF782	KS372550
		ZNF782	KS372551
		ZNF782	KS372552
		TET1	KS372553
	7 w	RPS20P12	KS372554
		AC144568	KS372555
		WAC-AS1	KS372556
**NL02.C**	1 h	NTM	KS372557
		NTM	KS372558
		NTM	KS372559
		NTM	KS372560

		NTM	KS372561
		NTM	KS372562
		NTM	KS372563
		NTM	KS372564
	24 h	L1	KS372565
		L1	KS372566
		L1	KS372567
		L1	KS372568
		L1	KS372569
		L1	KS372570
	3 d	L1	KS372571
		L1	KS372572
	2 w	NBPF25P	KS372573
		ZBED3	KS372574
**NL03.E**	7 d	S3A-37	KS372575
	2 w	HSAT-II	KS372576
		HSAT-II	KS372577
		HSAT-II	KS372578
		HSAT-II	KS372579
		HSAT-II	KS372580
		HSAT-II	KS372581
		HSAT-II	KS372582
		HSAT-II	KS372583
		HSAT-II	KS372584
		HSAT-II	KS372585
		HSAT-II	KS372586
		HSAT-II	KS372587

h, hour; d, day; w, week

SI-Table 2. GenBank accession numbers for WHV-woodchuck junctions identified in this study

Animal/Liver biopsy number	Time post-infection	Gene	Accession number
**Cw1/M - LBx 2**	1 h	MAML2	KS372114
		UI	KS372115
		EFTUD1P1	KS372116
**Cw2/F - LBx 2**	1 h	AAK1	KS372117
		AAK1	KS372118
		KIAA1117	KS372119
		LPIN3	KS372120
		PHACTR3	KS372121
**Cw4/M - LBx 2**	3 h	UI	KS372122
		UI	KS372123
		UI	KS372124
**Cw1/M - LBx 3**	6 w	UI	KS372125
		NLRC5	KS372126
**Cw2/F - LBx 3**	6 w	UI	KS372127
		UI	KS372128
**Cw3/F - LBx 3**	6 w	UI	KS372129
**Cw4/M - LBx 3**	6 w	UI	KS372130
		HDAC-9	KS372131

h, hour; w, week
